# Supplementary figures and images for: Predicting changes in protein thermodynamic stability upon point mutation with deep 3D convolutional neural networks
Source: PLoS Comput Biol. 2020 Nov 30;16(11):e1008291. doi: 10.1371/journal.pcbi.1008291 (PMC7728386; doi:10.1371/journal.pcbi.1008291)

(A)

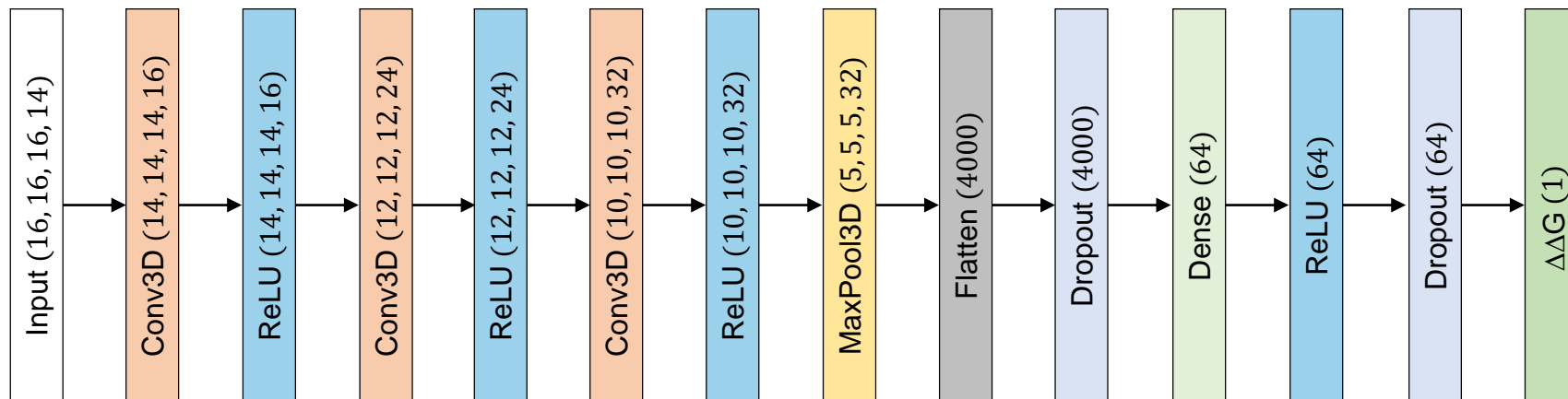

(B)

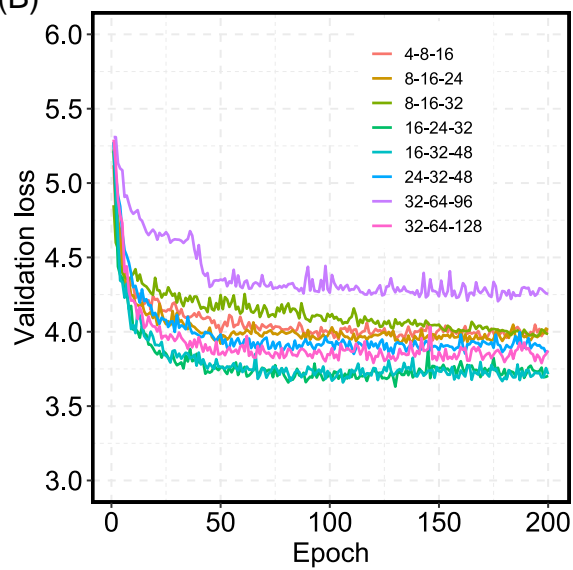

(C)

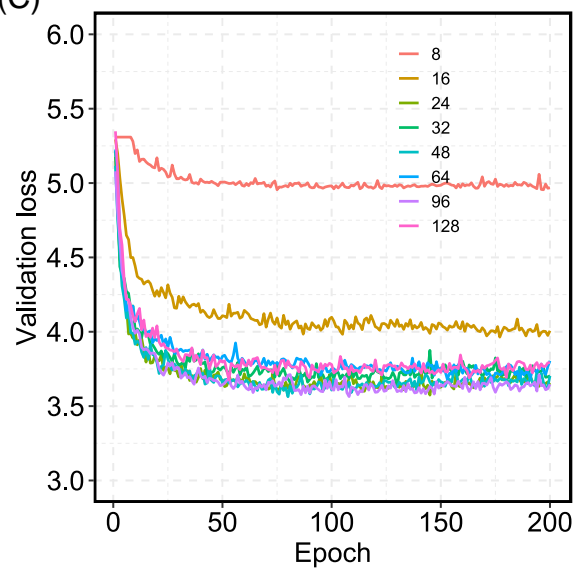

(D)

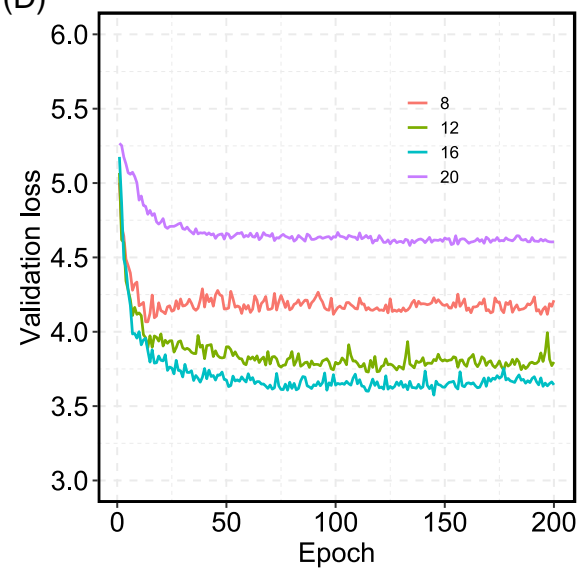

Supplement: S1 Fig — (A) The overall organization of the model begins with the input tensor and ends with a final layer that outputs ΔΔG prediction. The numbers in parentheses before the Flatten layer represent the dimensionality of the output from each layer in the format (width, height, depth, property channels). The number in parentheses starting from the Flatten layer represent the number of output features from each of the densely connected layers. This optimized architecture was determined through cross-validation. (B) Results from cross validating the sizes of the convolutional layers while keeping the size of the densely connected layer at 32 neurons. (C) Results from cross validating the size of the densely connected layer while keeping the sizes of the convolutional layers at (16, 24, 32). (D) Results from cross validating the dimensions of the input grid. (PDF) [file pcbi.1008291.s001.pdf]

(A) S2648 protein pairwise sequence identity

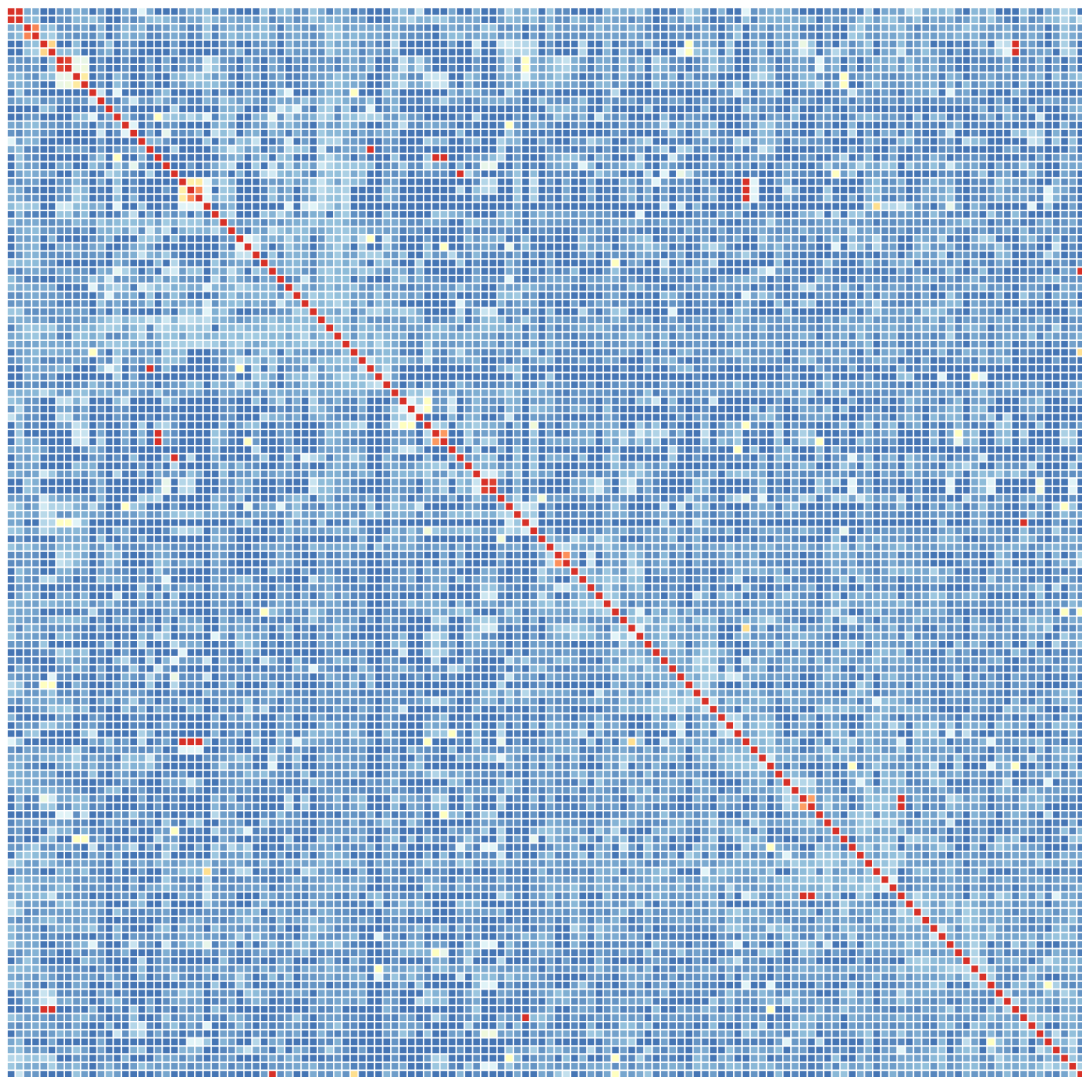

(B) VariBench protein pairwise sequence identity

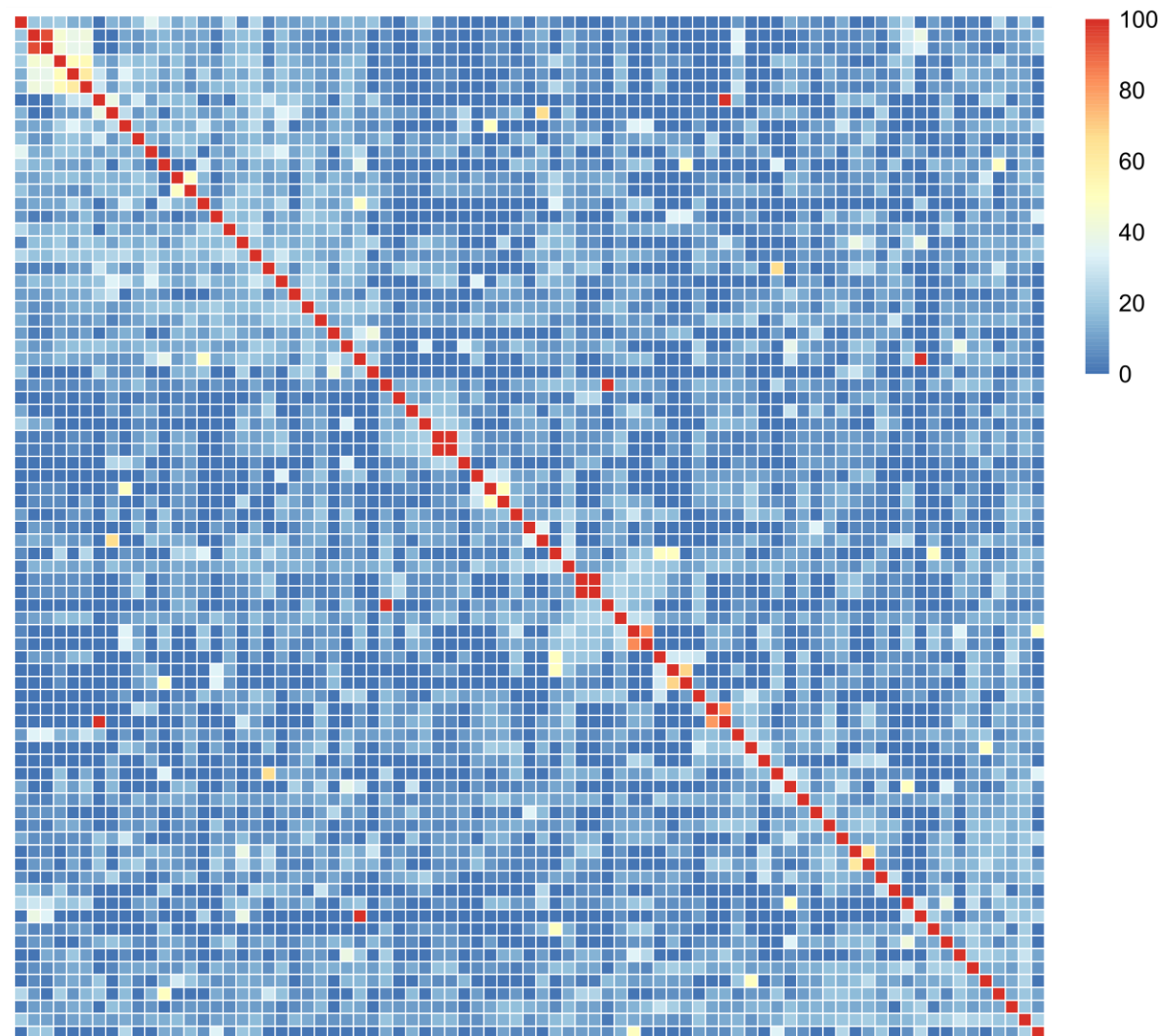

Supplement: S2 Fig — (A) A heatmap representation of the pairwise percent sequence identity matrix of the proteins in the S2648 data set. (B) A heatmap representation of the pairwise percent sequence identity matrix of the proteins in the VariBench data set. It is obvious from these two heatmaps that there is substantial pairwise homology (percent identity > 25%) in both S2648 and VariBench. The pairwise identity matrices were obtained using the Clustal Omega multiple sequence alignment program [73]. (PDF) [file pcbi.1008291.s002.pdf]

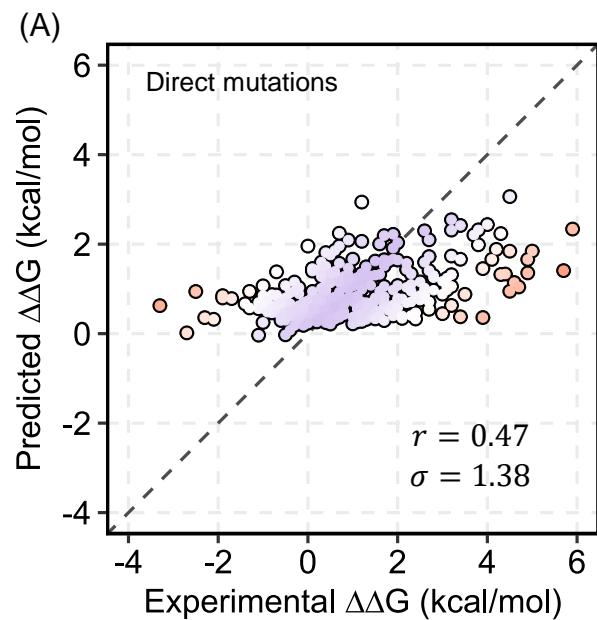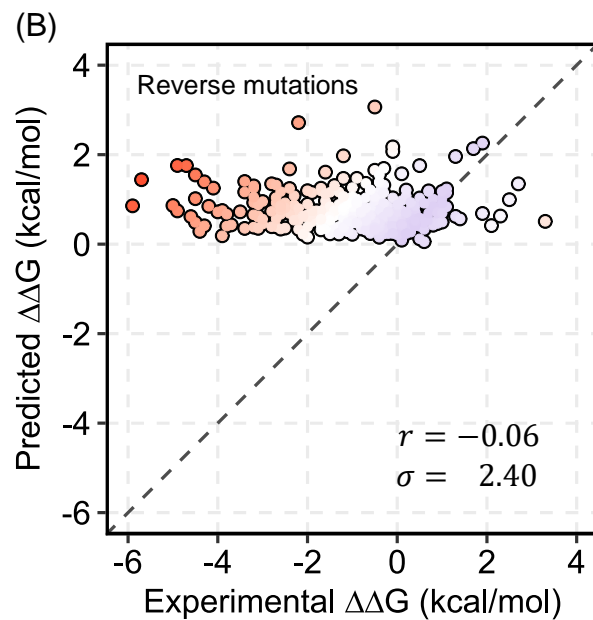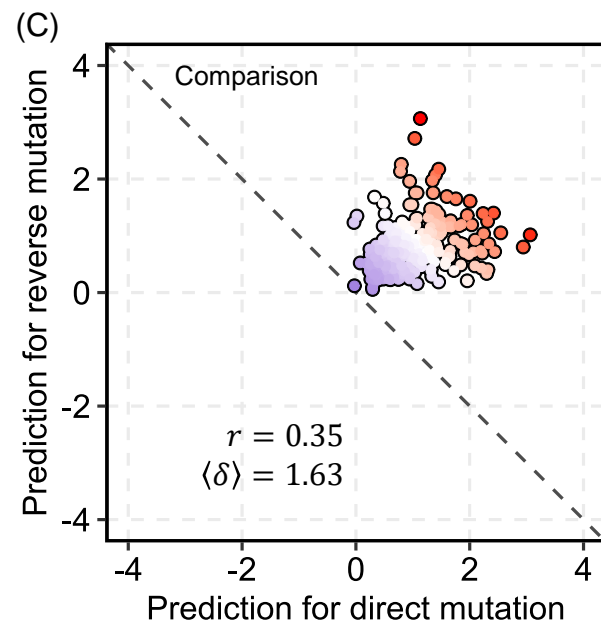

Supplement: S3 Fig — (A) Performance of an ensemble of ten networks trained using only the set of 1,744 direct mutations on predicting the ΔΔGs of the direct mutations in the blind test set; The Pearson correlation coefficient (r) between predicted values and experimentally determined values is 0.47, and the root-mean-square deviation (σ) of predicted values from experimentally determined values is 1.38 kcal/mol. (B) Performance of the same ensemble of ten networks on predicting the ΔΔGs of the reverse mutations in the blind test set; The Pearson correlation coefficient (r) between predicted values and experimentally determined values is -0.06, and the root-mean-square deviation (σ) of predicted values from experimentally determined values is 2.40 kcal/mol. (C) Direct versus reverse ΔΔG values of all the mutations in the blind test set predicted by the same ensemble of networks. (B) and (C) highlight that the models trained with only direct mutations have a large bias and, when compared to the models trained using the balanced data set, the necessity of adding reverse mutations to correct the bias. The dots are colored in gradient from blue to red such that blue represents the most accurate prediction and red represents the least accurate. (PDF) [file pcbi.1008291.s003.pdf]
