# Supplementary material for: Predicting changes in protein thermodynamic stability upon point mutation with deep 3D convolutional neural networks
Source: PLoS Comput Biol. 2020 Nov 30;16(11):e1008291. doi: 10.1371/journal.pcbi.1008291 (PMC7728386; doi:10.1371/journal.pcbi.1008291)
Supplement: S1 Table — (DOCX) [file pcbi.1008291.s004.docx]

S1 Table. PDB IDs of Identical proteins between PoPMuSiC-2.0 training and test sets.

| 1aj3A | 1cunA | 1hmkA | 1mjcA | 1titA | 2nvhA |
| --- | --- | --- | --- | --- | --- |
| 1akyA | 1dktA | 1hmsA | 1msiA | 1ttqA | 2rn2A |
| 1aonU | 1e65A | 1ifcA | 1oiaA | 1uzcA | 2trxA |
| 1apsA | 1ey0A | 1igvA | 1p2pA | 1yyjA | 3glyA |
| 1bniA | 1fnaA | 1ihbA | 1qlpA | 1zg4A | 3mbpA |
| 1btaA | 1ftgA | 1imqA | 1rg8A | 1znjB | 3pgkA |
| 1bvcA | 1g4iA | 1k9qA | 1risA | 2a36A | 3silA |
| 1c9oA | 1h7mA | 1lniA | 1rn1C | 2driA | 4lyzA |
| 1ceyA | 1hfzA | 1lz1A | 1rtbA | 2immA | 5dfrA |
| 1cseI | 1hmeA | 1mgrA | 1shfA | 2lzmA | 5ptiA |
| 1cspA |  |  |  |  |  |
