# Supplementary material for: Predicting changes in protein thermodynamic stability upon point mutation with deep 3D convolutional neural networks
Source: PLoS Comput Biol. 2020 Nov 30;16(11):e1008291. doi: 10.1371/journal.pcbi.1008291 (PMC7728386; doi:10.1371/journal.pcbi.1008291)
Supplement: S2 Table — (DOCX) [file pcbi.1008291.s005.docx]

S2 Table. PDB IDs of non-redundant proteins returned by submitting proteins in the S2648 data set to the PISCES server.

| 1a43A | 1dktA | 1jiwI | 1qlpA | 1yyjA |
| --- | --- | --- | --- | --- |
| 1aepA | 1e65A | 1k9qA | 1qm4A | 1zg4A |
| 1ag2A | 1ey0A | 1kcqA | 1qndA | 2a36A |
| 1akyA | 1fkjA | 1kdxA | 1rg8A | 2abdA |
| 1am7A | 1fnaA | 1ke4A | 1rhgA | 2driA |
| 1amqA | 1ftgA | 1kfwA | 1risA | 2lzmA |
| 1aonU | 1fvkA | 1lbiA | 1rn1C | 2nvhA |
| 1apsA | 1g4iA | 1lniA | 1ropA | 2ocjA |
| 1arrA | 1g6nA | 1lucA | 1sakA | 2rn2A |
| 1azpA | 1h7mA | 1lveA | 1shgA | 2trtA |
| 1b26A | 1hk0X | 1lz1A | 1supA | 2trxA |
| 1b8eA | 1hmeA | 1mbgA | 1tenA | 2ts1A |
| 1boyA | 1htiA | 1msiA | 1titA | 3ecaA |
| 1btaA | 1huuA | 1n0jA | 1tpkA | 3glyA |
| 1bvcA | 1ietA | 1oh0A | 1ttqA | 3hhrA |
| 1c9oA | 1ifcA | 1oiaA | 1tyvA | 3mbpA |
| 1cahA | 1igvA | 1oncA | 1ubqA | 3silA |
| 1ceyA | 1ihbA | 1pdoA | 1uzcA | 3ssiA |
| 1chkA | 1imqA | 1pgaA | 1vqbA | 5croA |
| 1cseI | 1io2A | 1pohA | 1witA | 5dfrA |
| 1cunA | 1iroA | 1qgvA | 1yu5X |  |
