# Supplementary material for: Predicting changes in protein thermodynamic stability upon point mutation with deep 3D convolutional neural networks
Source: PLoS Comput Biol. 2020 Nov 30;16(11):e1008291. doi: 10.1371/journal.pcbi.1008291 (PMC7728386; doi:10.1371/journal.pcbi.1008291)
Supplement: S4 Table — (DOCX) [file pcbi.1008291.s007.docx]

S4 Table. Proteins in the VariBench data set (Subject) that are either identical or likely to be homologous to proteins in the S^sym^ data set (Query).

| Query | Subject | %ID | Length | # Mismatches | Query start | Query end | Subject start | Subject end | E-value |
| --- | --- | --- | --- | --- | --- | --- | --- | --- | --- |
| 1bniA | 1bniA | 100 | 108 | 0 | 1 | 108 | 1 | 108 | 1.10E-80 |
| 1bniA | 1mgrA | 37.3 | 59 | 35 | 51 | 107 | 35 | 93 | 7.30E-07 |
| 1bniA | 1rggA | 33.8 | 80 | 44 | 31 | 107 | 19 | 92 | 6.51E-04 |
| 1ey0A | 1stnA | 100 | 136 | 0 | 1 | 136 | 1 | 136 | 5.08E-102 |
| 1iobA | 1iobA | 100 | 153 | 0 | 1 | 153 | 1 | 153 | 5.01E-116 |
| 1l63A | 1l63A | 100 | 162 | 0 | 1 | 162 | 1 | 162 | 1.75E-122 |
| 1l63A | 2lzmA | 98.8 | 162 | 2 | 1 | 162 | 1 | 162 | 1.94E-121 |
| 1lz1A | 1lz1A | 100 | 130 | 0 | 1 | 130 | 1 | 130 | 9.70E-98 |
| 1lz1A | 4lyzA | 60.9 | 128 | 49 | 1 | 128 | 1 | 127 | 5.73E-57 |
| 1lz1A | 1el1A | 52.3 | 130 | 61 | 1 | 130 | 2 | 130 | 5.53E-53 |
| 1lz1A | 1hfzA | 39.3 | 117 | 66 | 3 | 119 | 4 | 115 | 5.47E-30 |
| 1lz1A | 1hfyA | 39.3 | 117 | 66 | 3 | 119 | 3 | 114 | 3.31E-29 |
| 1rn1C | 1rn1B | 100 | 104 | 0 | 1 | 104 | 1 | 104 | 1.51E-76 |
| 1vqbA | 1vqbA | 100 | 86 | 0 | 1 | 86 | 1 | 86 | 2.31E-63 |
| 2lzmA | 2lzmA | 100 | 164 | 0 | 1 | 164 | 1 | 164 | 3.07E-125 |
| 2lzmA | 1l63A | 98.8 | 162 | 2 | 1 | 162 | 1 | 162 | 1.96E-121 |
| 2rn2A | 2rn2A | 100 | 155 | 0 | 1 | 155 | 1 | 155 | 6.59E-120 |
| 4lyzA | 4lyzA | 100 | 129 | 0 | 1 | 129 | 1 | 129 | 2.42E-96 |
| 4lyzA | 1lz1A | 60.9 | 128 | 49 | 1 | 127 | 1 | 128 | 5.68E-57 |
| 4lyzA | 1el1A | 53.1 | 130 | 59 | 1 | 129 | 2 | 130 | 1.51E-50 |
| 4lyzA | 1hfyA | 44.2 | 113 | 59 | 3 | 115 | 3 | 111 | 8.70E-31 |
| 4lyzA | 1hfzA | 41.6 | 113 | 62 | 3 | 115 | 4 | 112 | 3.22E-28 |
| 5ptiA | 1bpiA | 100 | 58 | 0 | 1 | 58 | 1 | 58 | 1.03E-41 |

Query represents proteins in the S^sym^ data set; Subject represents proteins in the VariBench data set; %ID is the percent identity of the alignment between the query sequence and the subject sequence; Query start/end and Subject end/end denote the starting and ending positions of the alignment in the query and subject sequences, respectively.
