# Supplementary material for: Predicting changes in protein thermodynamic stability upon point mutation with deep 3D convolutional neural networks
Source: PLoS Comput Biol. 2020 Nov 30;16(11):e1008291. doi: 10.1371/journal.pcbi.1008291 (PMC7728386; doi:10.1371/journal.pcbi.1008291)
Supplement: S5 Table — (DOCX) [file pcbi.1008291.s008.docx]

S5 Table. Proteins in the Q3421 data set (Subject) that are either identical or likely to be homologous to proteins in the S^sym^ data set (Query).

| Query | Subject | %ID | Length | # Mismatches | Query start | Query end | Subject start | Subject end | E-value |
| --- | --- | --- | --- | --- | --- | --- | --- | --- | --- |
| 1amqA | 1amqA | 100 | 396 | 0 | 1 | 396 | 1 | 396 | 0 |
| 1bniA | 1bniA | 100 | 108 | 0 | 1 | 108 | 1 | 108 | 2.21E-80 |
| 1ceyA | 1ceyA | 100 | 128 | 0 | 1 | 128 | 1 | 128 | 8.93E-93 |
| 1ey0A | 1stnA | 100 | 136 | 0 | 1 | 136 | 1 | 136 | 1.03E-101 |
| 1ihbA | 1ihbA | 100 | 156 | 0 | 1 | 156 | 1 | 156 | 4.49E-115 |
| 1ihbA | 1a5eA | 39.9 | 133 | 79 | 5 | 136 | 16 | 148 | 2.69E-26 |
| 1iobA | 1iobA | 100 | 153 | 0 | 1 | 153 | 1 | 153 | 1.01E-115 |
| 1l63A | 1l63A | 100 | 162 | 0 | 1 | 162 | 1 | 162 | 3.53E-122 |
| 1l63A | 2lzmA | 98.8 | 162 | 2 | 1 | 162 | 1 | 162 | 3.92E-121 |
| 1lz1A | 1lz1A | 100 | 130 | 0 | 1 | 130 | 1 | 130 | 1.96E-97 |
| 1lz1A | 4lyzA | 60.9 | 128 | 49 | 1 | 128 | 1 | 127 | 1.16E-56 |
| 1lz1A | 1el1A | 52.3 | 130 | 61 | 1 | 130 | 2 | 130 | 1.12E-52 |
| 1lz1A | 1hfyA | 39.3 | 117 | 66 | 3 | 119 | 3 | 114 | 6.67E-29 |
| 1oh0A | 1oh0A | 100 | 125 | 0 | 1 | 125 | 1 | 125 | 1.6E-94 |
| 1rn1C | 1rn1A | 99.0 | 104 | 0 | 1 | 104 | 1 | 103 | 1.16E-73 |
| 1vqbA | 1vqbA | 100 | 86 | 0 | 1 | 86 | 1 | 86 | 4.66E-63 |
| 2lzmA | 2lzmA | 100 | 164 | 0 | 1 | 164 | 1 | 164 | 6.19E-125 |
| 2lzmA | 1l63A | 98.8 | 162 | 2 | 1 | 162 | 1 | 162 | 3.96E-121 |
| 2rn2A | 2rn2A | 100 | 155 | 0 | 1 | 155 | 1 | 155 | 1.33E-119 |
| 4lyzA | 4lyzA | 100 | 129 | 0 | 1 | 129 | 1 | 129 | 4.89E-96 |
| 4lyzA | 1lz1A | 60.9 | 128 | 49 | 1 | 127 | 1 | 128 | 1.15E-56 |
| 4lyzA | 1el1A | 53.0 | 130 | 59 | 1 | 129 | 2 | 130 | 3.05E-50 |
| 4lyzA | 1hfyA | 44.2 | 113 | 59 | 3 | 115 | 3 | 111 | 1.76E-30 |
| 5ptiA | 1bpiA | 100 | 58 | 0 | 1 | 58 | 1 | 58 | 2.08E-41 |

Query represents proteins in the S^sym^ data set; Subject represents proteins in the Q3421 data set; %ID is the percent identity of the alignment between the query sequence and the subject sequence; Query start/end and Subject end/end denote the starting and ending positions of the alignment in the query and subject sequences, respectively.
