# Supplementary material for: Predicting changes in protein thermodynamic stability upon point mutation with deep 3D convolutional neural networks
Source: PLoS Comput Biol. 2020 Nov 30;16(11):e1008291. doi: 10.1371/journal.pcbi.1008291 (PMC7728386; doi:10.1371/journal.pcbi.1008291)
Supplement: S6 Table — (DOCX) [file pcbi.1008291.s009.docx]

S6 Table. A brief summary of the characteristics of methods presented in Table 2.

| Method | Algorithm | Feature | Reference |
| --- | --- | --- | --- |
| DDGun3D | Linear parametric model | BLOSUM62 substitution matrix, statistical potentials, hydrophobicity, solvent accessibility, and evolutionary information derived from multiple sequence alignment | [1] |
| DDGun | Linear parametric model | BLOSUM62 substitution matrix, statistical potentials, hydrophobicity, and evolutionary information derived from multiple sequence alignment | [1] |
| PoPMuSiC^sym^ | Same as PoPMuSiC 2.1 except two coefficients are constrained | Statistical potentials, amino acid volume | [2] |
| MAESTRO | Linear regression, neural network, and support vector machine | Statistical potentials | [3] |
| FoldX | NA | Empirical force field | [4] |
| PoPMuSiC 2.1 | Linear parametric model with coefficients fitted by a neural network | Statistical potentials, amino acid volume | [5] |
| SDM | NA | Environment-specific amino acid substitution table | [6] |
| iSTABLE | Meta predictor | Evolutionary information and predictions from I-Mutant, AUTOMUTE, MUPRO, PoPMuSiC, and CUPSAT | [7] |
| I-Mutant 3.0 | Support vector machine | Protein sequence and structure information | [8] |
| NeEMO | Neural network | Features extracted from residue interaction networks | [9] |
| DUET | Support vector machine | Predictions from mCSM and SDM | [10] |
| mCSM | Gaussian progress regression and random forest | Graph-based distance patterns | [11] |
| MUPRO | Support vector machine | Sequence information | [12] |
| STRUM | Gradient boosting regression | Sequence information, structure information, and evolutionary information derived from multiple sequence alignment | [13] |
| Rosetta | NA | Empirical energy function | [14] |
| AUTOMUTE | Support vector machine and random forest | Statistical potentials | [15] |
| CUPSAT | NA | Statistical potentials | [16] |

NA indicates either no machine-learning algorithm or no statistical model was used.

**References**

1. Montanucci, L., et al., *DDGun: an untrained method for the prediction of protein stability changes upon single and multiple point variations.* Bmc Bioinformatics, 2019. **20**(1).

2. Pucci, F., et al., *Symmetry Principles in Optimization Problems: an application to Protein Stability Prediction.* IFAC-PapersOnLine, 2015. **48**(1): p. 458-463.

3. Laimer, J., et al., *MAESTROweb: a web server for structure-based protein stability prediction.* Bioinformatics, 2016. **32**(9): p. 1414-6.

4. Guerois, R., J.E. Nielsen, and L. Serrano, *Predicting changes in the stability of proteins and protein complexes: A study of more than 1000 mutations.* Journal of Molecular Biology, 2002. **320**(2): p. 369-387.

5. Dehouck, Y., et al., *Fast and accurate predictions of protein stability changes upon mutations using statistical potentials and neural networks: PoPMuSiC-2.0.* Bioinformatics, 2009. **25**(19): p. 2537-2543.

6. Worth, C.L., R. Preissner, and T.L. Blundell, *SDM-a server for predicting effects of mutations on protein stability and malfunction.* Nucleic Acids Research, 2011. **39**: p. W215-W222.

7. Chen, C.W., J. Lin, and Y.W. Chu, *iStable: off-the-shelf predictor integration for predicting protein stability changes.* BMC Bioinformatics, 2013. **14 Suppl 2**: p. S5.

8. Capriotti, E., P. Fariselli, and R. Casadio, *I-Mutant2.0: predicting stability changes upon mutation from the protein sequence or structure.* Nucleic Acids Research, 2005. **33**: p. W306-W310.

9. Giollo, M., et al., *NeEMO: a method using residue interaction networks to improve prediction of protein stability upon mutation.* BMC Genomics, 2014. **15 Suppl 4**: p. S7.

10. Pires, D.E., D.B. Ascher, and T.L. Blundell, *DUET: a server for predicting effects of mutations on protein stability using an integrated computational approach.* Nucleic Acids Res, 2014. **42**(Web Server issue): p. W314-9.

11. Pires, D.E.V., D.B. Ascher, and T.L. Blundell, *mCSM: predicting the effects of mutations in proteins using graph-based signatures.* Bioinformatics, 2014. **30**(3): p. 335-342.

12. Cheng, J., A. Randall, and P. Baldi, *Prediction of protein stability changes for single-site mutations using support vector machines.* Proteins, 2006. **62**(4): p. 1125-32.

13. Quan, L.J., Q. Lv, and Y. Zhang, *STRUM: structure-based prediction of protein stability changes upon single-point mutation.* Bioinformatics, 2016. **32**(19): p. 2936-2946.

14. Simons, K.T., et al., *Assembly of protein tertiary structures from fragments with similar local sequences using simulated annealing and Bayesian scoring functions.* J Mol Biol, 1997. **268**(1): p. 209-25.

15. Masso, M. and I.I. Vaisman, *Accurate prediction of stability changes in protein mutants by combining machine learning with structure based computational mutagenesis.* Bioinformatics, 2008. **24**(18): p. 2002-2009.

16. Parthiban, V., M.M. Gromiha, and D. Schomburg, *CUPSAT: prediction of protein stability upon point mutations.* Nucleic Acids Research, 2006. **34**: p. W239-W242.
