# Supplementary material for: Predicting changes in protein thermodynamic stability upon point mutation with deep 3D convolutional neural networks
Source: PLoS Comput Biol. 2020 Nov 30;16(11):e1008291. doi: 10.1371/journal.pcbi.1008291 (PMC7728386; doi:10.1371/journal.pcbi.1008291)
Supplement: S7 Table — (DOCX) [file pcbi.1008291.s010.docx]

S7 Table. Comparison of ThermoNet with four other methods on p53.

| Method | $\boldsymbol{\sigma}_{\boldsymbol{dir}}$ | $\mathbf{r}_{\boldsymbol{dir}}$ | $\boldsymbol{\sigma}_{\boldsymbol{rev}}$ | $\mathbf{r}_{\boldsymbol{rev}}$ | $\mathbf{r}_{\boldsymbol{dir-rev}}$ | $\left\langle\boldsymbol{\delta} \right\rangle$ |
| --- | --- | --- | --- | --- | --- | --- |
| FoldX | 1.79 | 0.71 | 1.51 | 0.75 | -0.97 | 0.23 |
| ThermoNet | 2.01 | 0.45 | 1.92 | 0.56 | -0.93 | -0.04 |
| Rosetta | 3.47* | 0.78 | 2.75* | 0.70 | -0.84 | 1.15 |
| SDM | 1.52 | 0.68 | 2.44 | 0.01 | -0.37 | 0.71 |
| CUPSAT | 2.83 | 0.25 | NA | NA | NA | NA |

NA: not available. The CUPSAT server does not offer batch processing for predicting the ∆∆Gs of reverse mutations nor a downloadable standalone version for local use.

* Rosetta’s large $\sigma$ values are due to a few mutations for which Rosetta had a large prediction error.
