# Supplementary material for: Predicting changes in protein thermodynamic stability upon point mutation with deep 3D convolutional neural networks
Source: PLoS Comput Biol. 2020 Nov 30;16(11):e1008291. doi: 10.1371/journal.pcbi.1008291 (PMC7728386; doi:10.1371/journal.pcbi.1008291)
Supplement: S8 Table — (DOCX) [file pcbi.1008291.s011.docx]

S8 Table. Comparison of ThermoNet with four other methods on myoglobin.

| Method | $\boldsymbol{\sigma}_{\boldsymbol{dir}}$ | $\mathbf{r}_{\boldsymbol{dir}}$ | $\boldsymbol{\sigma}_{\boldsymbol{rev}}$ | $\mathbf{r}_{\boldsymbol{rev}}$ | $\mathbf{r}_{\boldsymbol{dir-rev}}$ | $\left\langle\boldsymbol{\delta} \right\rangle$ |
| --- | --- | --- | --- | --- | --- | --- |
| FoldX | 1.40 | 0.61 | 1.36 | 0.60 | -0.97 | 0.12 |
| ThermoNet | 1.16 | 0.38 | 1.18 | 0.37 | -0.97 | -0.02 |
| Rosetta | 4.72* | 0.63 | 3.62* | 0.63 | -0.93 | 0.91 |
| SDM | 1.25 | 0.52 | 1.48 | 0.12 | 0.18 | 0.89 |
| CUPSAT | 1.60 | 0.25 | NA | NA | NA | NA |

NA: not available. The CUPSAT server does not offer batch processing for predicting the ∆∆Gs of reverse mutations nor a downloadable standalone version for local use.

* Rosetta’s large $\sigma$ values are due to a few mutations for which Rosetta had a large prediction error.
